# Understanding the family caregivers’ needs in late-stage dementia: a qualitative perspective to inform policy action

**DOI:** 10.1186/s12877-026-07201-7

**Published:** 2026-03-10

**Authors:** Silvia Gonella, Paola Di Giulio, Jacopo Maria Olagnero, Daniele Quaranta, Giulia Nagliati, Valerio Dimonte, Sara Campagna

**Affiliations:** 1 Department of Public Health and Pediatrics, University of Torino, Santena street 5 bis, Turin, 10126 Italy; 2 Direction of Health Professions, City of Health and Science, University Hospital of Torino, Corso Bramante 88-90, Turin, 10126 Italy

**Keywords:** Caregivers, Caregiver burden, Dementia, Needs assessment, Public policy, Qualitative research

## Abstract

**Background:**

Dementia places substantial burden on family caregivers due to its irreversible and progressive nature. Prior research mainly explored family caregivers’ needs in the early- and middle-stage of dementia. However, as dementia progresses, caregiving demands intensify and caregivers’ needs evolve, often contributing to nursing home (NH) admission. Assessing needs across the disease trajectory is essential for designing tailored interventions. This study aimed to explore family caregivers’ self-perceived needs in late-stage dementia, primarily in the NH context and during the transition into NH.

**Methods:**

A qualitative descriptive study was performed. Data were collected via semi-structured in-depth interviews with open-ended questions and then analyzed with inductive content analyses. Thirteen family caregivers of people with advanced dementia were interviewed. They were purposively identified by the NH managers (*n* = 11) and the local palliative care service (*n* = 2).

**Results:**

Family caregivers had information, psychosocial, and self-care needs that were deeply intertwined and frequently coexisted at the same time. Information needs concerned clinical issues including disease trajectory and treatment options, legal issues such as the role of family caregivers in the decision making-process, bureaucratic issues including State assistance and benefits, and information on the available services in the community. They looked for formal care providers’ support to satisfy their information and psychosocial needs, while they relied on informal support for obtaining emotional support, releasing stress, and managing daily care duties.

**Conclusions:**

Our study findings confirm that NH placement does not completely relieve caregiving burden and highlight the recurrence of well-known needs that can be targeted through interventions aimed at promoting dementia understanding and services knowledge, or ensuring psychosocial or material support in daily care. In all, these findings call for moving from needs assessment to actionable interventions. A mapping of the resources available in the community can be the starting point for facilitating networking to answer timely and effectively to dementia family caregivers’ care needs, and appears as a useful aid to inform public health planning and resource allocation.

**Supplementary Information:**

The online version contains supplementary material available at 10.1186/s12877-026-07201-7.

## Background

Dementia is one of the most prevalent and challenging non-communicable diseases within the global population with significant disability and dependency among older individuals. More than 55 million people live with dementia worldwide and every year there are nearly 10 million new cases [1]. Only in Europe, the number of people with dementia is estimated to be 7.8 million and is expected to nearly double by 2050 [2].

Dementia is irreversible and progressive in nature with a long duration and family members usually assume the role of informal caregiver [3, 4]. The Alzheimer Association estimates that in 2023 more than 11 million family members and other unpaid caregivers provided 18.4 billion hours of care that indicates a decline in the number of caregivers compared to a decade ago, along with an increase in the amount of care by each remaining caregiver [5]. This negatively affects their quality of life and most dementia caregivers report several needs across diverse domains including physical and mental health, social, legal and financial affairs [6]. As the disease progresses and the caregivers’ burden increases, detrimental changes in their health-related outcomes and cognitive function occur [7, 8]. Over two-thirds of dementia caregivers report needs related to their caregiving role [6, 9] that may impact the quality of care they provide and the timing of nursing home (NH) placement, in addition to their well-being [10].

Transitioning into NH becomes a frequent choice to relieve the caregiving burden as the physical functioning worsens and the management of behavioral problems becomes more complex [11]. However, NH placement does not entirely relieve the burden of caregivers’ responsibilities, as they undertake different duties, such as monitoring the quality of care their relative receives [12]. Moreover, programs designed to engage family caregivers in interventions aimed at improving care quality require them to squeeze extra activities in their family and working schedule [13]. Overall, for family caregivers NH admission represents a challenging transition that requires information and emotional support to adapt to the new role [14].

Regardless of the care setting, support for dementia caregivers represents one of the priorities of the global action plan on the public health response to dementia 2017–2025 of the World Health Organization to allow them living a life with meaning and dignity [15]. As dementia progresses to advanced stages, caregiving demands and needs can evolve accordingly. Late-stage dementia is marked by functional decline, complex symptom management and difficulty in communication, and requires family caregivers to navigate difficult decisions including transitions in care settings, coordinate care services and reorganise daily life accordingly [16]. These circumstances can generate unique needs during a period of increased workload and role strain.

Unmet needs are areas where support is insufficient, inadequate, or unavailable [17]. Assessing unmet needs in people with dementia and their caregivers represents the first step to develop targeted interventions [9, 16, 18, 19]. However, prior research has mainly focused on the early- and middle-stage of the illness [18, 19], while the perspective of family caregivers engaged in the care of people with advanced dementia (PwAD) has been poorly investigated [16]. Moreover, little is known about how needs are experienced in the NH setting and during transition into NH, when caregiving responsibilities change.

Gaining a comprehensive understanding of dementia family caregivers’ needs at each transition across disease severity and care settings, it is pivotal for designing tailored interventions that can effectively support them in their caregiver role [20]. Therefore, the purpose of this study was to explore family caregivers’ self-perceived needs in late-stage dementia, primarily in the NH context and during the transition into NH.

## Materials and methods

### Study design

A qualitative descriptive approach with inductive content analysis [21] was employed to highlight experiences of family caregivers of PwAD still alive or recently deceased. Qualitative description aims to understand the phenomena and the subjects involved. Its defining feature is a close adherence to data with the absence of an interpretative description, which is instead characterized by a high degree of abstraction or conceptualization [22].

The Consolidated Criteria for Reporting Qualitative studies guidelines were followed (Appendix 1) [23].

### Setting and sample selection

Family caregivers of PwAD who lived in Northern Italy were identified through NH managers, the local palliative care service, and two local peer support groups for dementia caregivers.

Family caregivers with any degree of kinship with the PwAD were deemed eligible to participate if: (a) they were actively engaged in their relative’s care, at home or in NH, based on referrers’ judgement; (b) their relative had a Functional Assessment Staging Tool (FAST) score ≥7 A (i.e., speech limited to approximately a half-dozen intelligible words or fewer over an average day) [24], or the life expectancy was < 6 months, or died from dementia three to twenty months prior to recruitment; and (c) were available to participate and provided written informed consent to audiotape their interview.

### Data collection

Two trained researchers conducted semi-structured in-depth interviews between April and September 2023, either in-person at participants’ convenient places or via telephone. Two literature-informed interview guides were developed [25, 26]: one for family caregivers whose loved one was still alive (Appendix 2) and one for bereaved family caregivers (Appendix 3). The interview guides explored: (a) changes in the PwAD health status; (b) family caregivers’ preparedness for their relative’s worsening; (c) family caregivers’ preparedness for what can happen in the coming months and decisions that would likely need to be made (only if the PwAD was still alive); and (d) knowledge of local associations and community services that provide support to PwAD and their family caregivers.

Interviews were digitally audio-recorded and had flexible duration.

Additional data on family caregivers’ characteristics (gender, age, education, employment status, relationship to the PwAD, care setting, current vs. bereaved caregiver status, and visiting frequency in NH if applicable), and PwAD characteristics (time since dementia diagnosis, length of NH stay, and time since if applicable) were collected using an interviewer-administered questionnaire.

The interview guide was pilot-tested with two family caregivers (data not included in the final dataset) with only minor changes to the final format.

### Transcription and data analysis

Two researchers transcribed the interview recordings verbatim and anonymized them. A third researcher checked the transcripts against the recordings for accuracy. An inductive content analysis based on familiarizing with the dataset, developing initial codes, clustering similar codes into categories, and similar categories in themes [21] was performed with the aid of the software Atlas.ti 8.

Two researchers independently developed an initial list of codes from the first three transcripts and met to agree on a consensus list. This was discussed and refined within the team and applied to the analysis of the remaining transcripts. An iterative process was followed with coding being repeatedly revised and transcripts recoded. Three researchers did the analysis to ensure inter-rater agreement and the entire team was engaged in constant discussion to strengthen the analytical rigor. Coding was conducted in Italian for close engagement with the data. Illustrative quotes were translated into English and identified by a code indicating the interviewee’s relationship to the PwAD, age, and care setting.

### Rigour

Guidelines for trustworthiness were followed [27]. Several strategies were implemented to ensure credibility and dependability. Semi-structured interviews provided an in-depth understanding of PwAD family caregivers’ needs and were conducted by two nurses who received additional training in qualitative interview techniques. Three researchers did the analysis by employing a consolidated coding framework that was based on two independent lists of codes. An audit trail was maintained by the research team. Triangulation within the team aided in identifying categories, significant quotes, and offering explanations and interpretations of the findings. These measures guaranteed confirmability. Transferability was ensured by detailing the data collection process, sample characteristics, and achieving data saturation.

### Ethical considerations

This study was approved by the Ethics Committee of the University of Torino (prot. 131362, 5 March 2020). Participation was voluntary and based on written informed consent. Trained researchers experienced in discussing sensitive topics conducted the interviews, and participants could choose the interview location.

Given the sensitive nature of discussing advanced dementia care, several measures were implemented to minimise distress [28]. To avoid approaching families during acute bereavement while also limiting recall bias, we planned to recruit bereaved caregivers within 3–20 months after the death. Moreover, all participants were offered the option to review the topic guide in advance, adequate time was allowed for responses, and questions were rephrased as needed.

A distress protocol was applied: interviewers monitored emotional cues and, if signs of distress emerged, paused the interview, offered immediate support and a break, and, if needed, discontinued the interview. Participants were encouraged to set the pace and depth of disclosure; when appropriate, the interviewer allowed brief deviations from the topic to acknowledge and accommodate challenging experiences. At the end of each interview, researchers provided information on available dementia support services. Finally, interviews were conducted in the participant’s preferred language to facilitate understanding and reduce discomfort.

## Results

### Participants

Overall, 203 NH managers were mailed the study protocol and approached by telephone and only 6 (3%) contributed to the study; two NH managers involved 11 family caregivers, while four NHs managers found no family caregivers available to participate. Two additional family caregivers were identified by the local palliative care service and none by the peer support groups.

In all, 13 family caregivers (8 daughter/son, 2 spouse, 2 nephew/niece, and one daughter in law), ten of which were females, participated in the project; 12 were current caregivers (11 in NH and one at home) and one (home caregiver) lost their relative 8 months prior to the interview. The mean age was 62 years (range 51–82) and the education level was mainly high school (*n* = 8), followed by middle school (*n* = 3), and degree (*n* = 2). Five were retired, four employed, two freelance, and two unemployed. The frequency of visits in NH varied: daily for one caregiver, three or four times a week for two, once or twice a week for five, or once a month or less for three. Two interviewees lived with the PwAD.

At the time of the interview, PwAD had their diagnosis, on average, 7.4 years (4–15 years) earlier and the mean NH stay was 4.4 years (range 1–12 years). Ten family caregivers had in-person interview (eight in NH and two at home) and three via telephone. The mean duration of interviews was 38 min (range: 21–80 min).

### Family caregivers’ self-perceived needs in late-stage dementia

Overall, eleven categories that describe the needs of PwAD family caregivers were identified and gathered in three themes: information needs, psychosocial needs, and self-care needs and life reorganization (Table 1).

**Table 1:** Family caregivers’ self-perceived needs in late-stage dementia

Themes	Categories
Information needs	Clinical issues
	Legal issues
	Bureaucratic issues
	Available services in the community
Psychosocial needs	Effective communication
	Professional support
	Peer support
Self-care needs and life reorganization	Care burden
	Life revolving around the person with dementia
	Human resources
	Organizations and volunteering associations

### Theme 1: Information needs

Family caregivers described information needs as a cross-cutting requirement that shaped how confidently they navigated caregiving decisions and daily care tasks. Four interrelated categories of information needs emerged: (1) clinical issues including disease trajectory, symptoms progression, and treatment options; (2) legal issues such as their role and responsibilities in the decision-making process or how to have legal arrangements in place; (3) bureaucratic issues related to accessing State assistance and benefits; and (4) available services in the community.

#### Information needs related to clinical issues

Most interviewees complained of poor information regarding disease progression, particularly for the deterioration of cognitive and motor functions, or the onset or escalation of behavioral symptoms.

Unmet clinical information needs frequently concerned practical management of everyday problems such as mobility, feeding, and constipation, and were especially relevant for family caregivers providing home care. Participants described a desire for anticipatory guidance that could help them prepare and respond appropriately as symptoms evolved.

*“When you come into this world* [of dementia caregiving], *someone should*,* I don’t say prepare you*,* because you’re never prepared anyway… but at least provide an idea of what might happen.”* (Daughter, 51y, NH).

*“If someone had told me ‘she is going to get worse and may become violent’… My mother was never violent and having to run away because she threw at me everything… I’d have had benefit from a little idea like “it can escalate to violence… or be careful because she can fall and get hurt twice as bad”.* (Daughter, 59y, NH)

*“I never thought that everything would become paralyzed*,* even almost the voice”.* (Spouse, 81y, home)

The options of care for people with late-stage dementia were rarely discussed with their family caregivers in clinical encounters. Only two interviewees recalled receiving information about palliative approaches for people with dementia, indicating a gap in awareness that may limit timely consideration of comfort-oriented care. One spouse explicitly described learning that “comfort care” could apply beyond cancer-related illness.

*“The physician proposed me to go with comfort care…otherwise I’d have continued to think that such care could only be done when one has a cancer”.* (Spouse, 82y, NH)

When looking for information on symptom management and care treatments, family caregivers usually search for open discussion with healthcare professionals (NH staff or general practitioners) and peers. Self-information via media was limited and mostly unsuccessfully.

*“I usually talk with the nurse who is the one who stays with my mum the most and knows her the best”.* (Daughter, 51y, NH)

#### Information needs related to legal issues

A significant number of interviewees doubted their role in the decision-making process. Also, the interviews emerged wide misinformation on legal authority in decision-making with almost all wrongly believing they could make decisions on behalf of their relative by virtue of kinship, even in the absence of any legal documents. Some interviewees described routinely signing documents despite the absence of legal authorization, while a daughter questioned whether being an only child automatically implied decision-making authority.

Of the 13 interviewees, only three reported having a formal legal arrangement in place (two held a power of attorney and one had a court-appointed full guardianship) and all expressed the need for more and clearer information on this topic. Some of them were considering formal decision-making supports in the future, depending on their relative’s evolving needs and capacity.

*“… as the only daughter*,* I thought the decisions were up to me. If this is not the case*,* it should be clarified to me.”* (Daughter, 52y, NH).

*“We have no legal documents in place but I’ve always signed because the others are not there. Someone needs to take responsibility*,* I’m the daughter*,* so I can do that.”* (Daughter, 59y, NH).

*“My mom can’t even find her feet anymore*,* so I think we may need to seek a court-appointed guardianship measure. […] This is something I’d like to understand better: how does the process work and what am I supposed to do?’”* (Daughter, 53y, NH).

#### Information needs related to bureaucratic issues

Most interviewees reported receiving minimal or no information regarding how to access State assistance (e.g. regional contribution to NH fees and attendance allowance) and benefits (e.g. parking permits and tax deductions for care-related expenses) in the initial stages of their relative’s illness. Several family caregivers complained of missing a single point of contact that provides comprehensive information on public assistance and benefits available. They reported a highly fragmented information process that required them to navigate the system independently with a negative impact on their well-being. They felt lost and overwhelmed by administrative steps when support was unavailable and linked this bureaucratic uncertainty to additional burden and emotional strain. Only three interviewees received such information from NH staff or acquaintances.

*“When this disease starts*,* you get lost if you don’t know how to get out of a little bit. You have to get the authorization for assistance*,* the parking permits… and all these things. If you don’t have anybody to help you… that’s why you become sick*,* because you don’t know what to do…”* (Spouse, 82y, home).

#### Information needs related to available services in the community

Family caregivers usually reported not being aware of local associations and community-based resources that offer support to people with dementia and their family caregivers. Only one home caregiver described relying on an association that offered peer support groups, guidance on legal protection measures, and recreational activities. However, most NH family caregivers would have sought community support (especially peer support groups) if they had been informed that such services existed. When available and known to caregivers, Alzheimer’s cafés and voluntary dementia care associations were useful in facilitating contact with professionals and providing guidance on guardianship-related matters.

*“I relied on an Alzheimer’s café and on Amacare* [voluntary association] … *professionals come there… I found the geriatrician who was really good and a lawyer who is helping me with the guardianship”.* (Spouse, 82y, home)

*“If I had known about these community services that support people with dementia and their family caregivers when my aunt was still at home*,* I would definitely have used them. But as things are now*,* I don’t think so. Now*,* if I need anything*,* I ask the nursing home staff directly”.* (Nephew, 51 years, NH)

### Theme 2: Psychosocial needs

Caregiving had a profound emotional impact on family caregivers who described the need for multi-layered psychosocial support to sustain their role over time. Support needs spanned both professional relationships and informal networks (peers, other family members, friends, peer support groups).

A key need concerned effective communication with professionals, particularly in the NH context. Family caregivers valued having a person of reference who could acknowledge concern, contain anxiety, and facilitates timely information. Prompt updates about health issues or changes in treatment plans was perceived as a form of inclusion in the decision process that strengthened trust in the care team and reduced concerns. Instead, they were dissatisfied when communication was perceived as reactive and they had to seek information, or when notifications about significant events occurred late.

*“There is a nurse in particular… she is very kind and available. I have her personal cell phone number*,* so at times I call her even if she is not on duty.”* (Daughter, 52y, NH).

*“I ask when I notice something out of the ordinary … they inform me when they take her to the emergency department*,* well*,* not the first time but the second one because I was pissed off. At least inform me* [laughs]… *in fact*,* the second time they called me.* (Nephew, 51y, NH).

Some family caregivers also reported a need for psychological support to manage distress, anticipatory grief, and emotional overload. Two interviewees regularly visited a psychologist. A few family caregivers requested psychological support from the hospital in the early stages of the illness but their need remained unmet; others would appreciate a community-based psychological support service that could provide listening support and guidance in addressing problems.

*“I asked if there was any meeting*,* something just to understand… how this disease works when my dad was cared for in the neurology department of Hospital X. I didn’t get any answer.”* (Daughter, 57y, NH).

*“It would take a psychologist*,* a service that cares for you… it would take a community desk that listens to your problems and solves them or at least guides you in solving them.”* (Spouse, 82y, home).

However, not all family caregivers felt they need psychological support, and preferred to deal with distress on their own:

*“I prefer to face my demons alone*,* I got over the sudden death of my husband*,* I do not need any psychological support.”* (Daughter, 73y, NH).

Peer support emerged as a meaningful resource, primarily through informal conversations with acquaintances or friends who had current or past experience of dementia care. Sharing experiences with peers was described as normalising and relieving, helping family caregivers anticipate what to expect and identifing practical ways forward with greater confidence and calm. However, in this sample, participation in structured support groups was rare; several interviewees were unaware such opportunities existed and indicated they would have participated if informed. Two family caregivers planned to serve as moderators in peer support groups in the future to support their peers.

*“We have several friends who share our experience*,* either with parents or siblings in nursing home or with home assistance. We started to understand that this thing happened and has to be faced with serenity”.* (Daughter-in-law, 54y, NH)

*“Even just to talk with people who already had this experience. Like when you’re an alcoholic. Figure out how to move*,* what to expect… it helps hearing from someone who has been through your experiences and they tell on them non-dramatically.”* (Daughter, 51y, NH).

### Theme 3: Self-care needs and life reorganization

Family caregivers complained of a progressive loss of their personal well-being and the need to reorganize everyday life around the person with dementia. Almost all family caregivers reported high levels of physical and psychological strain with poor appetite, insomnia, anxiety and depression, particularly when caring for the relative at home. Stress was intensified by disease progression, behavioral symptoms, the increasing difficulty of communication with their relative, and a persistent sense of being “on duty”, with little or no respite and leisure time. As a result, family caregivers frequently deprioritised their own needs, and, in some cases, modified or disrupted work routines. One daughter framed this as a pervasive bodily and emotional involvement, while another described the long-term cumulative nature of this strain and emphasized the absence of breaks and holidays as a key mechanism of exhaustion.

*“I didn’t eat anything*,* I gained weight*,* I don’t know. I don’t know if it’s the nervousness… in the end you are involved from head to toe.”* (Daughter, 57y, NH).

*“It has been a hallucinating period*,* we were exhausted*,* we really reached the end of our rope*,* also because you can never move*,* never go on vacation*,* you can’t do anything. And after so many years it wears you down.”* (Daughter, 53y, NH).

A central need underpinning these accounts was practical support in daily care duties while the relative was living at home. Participants usually relied on informal networks (other family members or friends) or paid family assistants. However, when support was insufficient or when family assistants were perceived as inadequately equipped to care for PwAD, the institutionalization became more likely. The decision to move the relative to a NH was often portrayed as constrained, and was accompanied by feelings of ambivalence and guilt.

*“My aunt is in a nursing home now*,* but if I’d had found two skilled family assistants*,* I could have stayed her at home. Either way*,* the costs are basically the same. The nursing home was a forced choice.”* (Niece, 51y, NH).

Following NH admission, several family caregivers reported a reduction in day-to-day burden. Many described regaining time for basic routines (sleep, personal hygiene) and, over time, resuming interests and activities that had been suspended. This transition involved a period of adjustment as family caregivers adapted to the new arrangement. One participant described this as a sudden confrontation with silence and regained sense of autonomy:

*“That day*,* on January 13*,* I sat on the couch for the first time and said to myself ‘what do I do now?’. I wasn’t used to all that silence*,* I could take a shower*,* go to sleep … I mean all those things I had never done! It’s ugly to say*,* however*,* I took my life in my hands…”* (Daughter, 51y, NH).

## Discussion

This qualitative descriptive study explored family caregivers’ experiences of their needs in late-stage dementia, primarily in the NH context and during the transition into NH. The main finding is that needs commonly described in early and middle stages -informational, psychosocial, and self-care- remain relevant and frequently unmet in late-stage dementia, even after NH placement. This confirms that NH admission should not be considered as a turning point and that information should be continuous and tailored on the evolving caregivers’ needs.

Our family caregivers often reported a variety of needs across several domains and many of them were interrelated, consistently with previous literature [9]. Home caregivers mainly described physical and mental health issues with unmet self-care needs. In line with dementia literature [29, 30], they reported high engagement in their relative’s activities of daily living with limited or no time for their personal life; this resulted in insomnia, poor appetite, anxiety and depressive symptoms. After NH placement, physical demands decreased, but emotional strain persisted. In this phase, the interviewees emphasized the importance of a reference person who facilitates timely updates, ensures continuity in communication, and accommodates their anxiety and uncertainty [31]. This suggests that transitions shape the type of caregiver’s burden rather than resolving it.

Family caregivers’ information needs were pivotal and strongly associated with their preparedness for end-of-life care planning and their understanding of their decision-making role. Consistently with previous studies [3, 32, 33], they wanted clear information on disease progression and behavioral and psychological symptoms of dementia, as well as information about how to navigate decisions as substitute decision makers. Literature shows that difficulties in understanding the likely dementia trajectory and how to interpret changes such as swallowing difficulties and recurrent infections negatively impact end-of-life care planning, family caregivers involvement in the decision-making process, and finally the quality of care provided with increase in burdensome intervention and distress near the end of life [34–36]. These findings align with the necessity to invest in communication skills training, particularly in the NH context, to improve communication on disease trajectory, what to expect as dementia progresses, care options, and the role of family caregivers in the decision-making process [37]. Such guidance is core for ensuring optimal care in dementia [38, 39], and when provided family caregivers report more positive perception of care and less decision-making uncertainty [40]. In all, our results support the need of a structured end-of-life communication process that begins before NH admission and continues after institutionalization, rather being deferred until acute deterioration.

Similarly to previous works [30], our interviews also emerged poor information of community-based services and associations that can support people with dementia and their caregivers, particularly at the initial stage of the disease. This information gap matters because missed information is likely to result into non-use of available support, caregiver’s physical and psychological exhaustion, and finally a forced decision to institutionalize [17, 41]. This is against the preference of most people who wish to remain at home as long as possible [42] and the recommendations to postpone NH admission due to potential negative impact on autonomy, daily routines, and social activities [43]. From a human rights-based perspective [44], limited access to information raises ethical concerns whether rights are effectively enabled. This ethical question becomes even more impelling when evidence shows that information and support are associated with lower psychological distress among family caregivers [45], and community-based services can delay NH entry [46].

Limited public awareness of dementia services is a well-known criticism of the Italian care system that has been documented over the past two decades [41]. However, initiatives to promote information about available services remain limited [47, 48], while research on needs assessment continues to grow and often reiterates similar findings [49]. Policymakers would therefore benefit more from implementation research aimed at supporting referral practice. Mapping services to know what is available may favor early and correct information, timely referral to the appropriate service and need satisfaction, and inform public health planning and resource allocation towards services that need to be newly established or others that would benefit from strengthening. The Italian National Dementia Plan recommends the mapping of healthcare and social services available, also with the collaboration of municipalities, as the starting point for facilitating networking and establish collaborations to answer care needs [50]. A comprehensive mapping should encompass third-sector organisations, considering their potential to complement public and private provision in satisfying the increasing social care demand of our ageing society [51].

Beyond a map of the services, already available in several countries/areas including Italy, an analysis of scopes, activities, and targeted audience of each service with involved healthcare professionals can improve the need-based guidance. Future research is needed to explore changes in the access to services and factors that influence their use, which could lead to more evidence-based policies.

The use of qualitative descriptive methodology and flexibility in interviews modalities to promote inclusivity and comfort ensure that findings closely reflect family caregivers’ needs in late-stage dementia, which can inform clinical practice and policy. However, the study had some limitations. Recruitment of family caregivers was challenging and it is possible that participating NHs were those more engaged in quality improvement and that family caregivers who agreed to participate had more positive experiences. This may have contributed to a more optimistic view of the needs identified. However, at the same time, these perspectives inform on practices that family caregivers perceived as supportive in the NH setting (e.g., single point of contact, proactive information sharing). Such findings may serve as a reference point to strengthen community- and home-based services, design interventions to better support family caregivers earlier in the disease trajectory, and potentially ease, delay, or even avoid transitions to institutional care. Moreover, home caregivers were poorly represented, even if this was quite expected since around two-thirds of people with dementia permanently enter NHs at some point in the disease trajectory because of increased dependency [52]. Finally, findings may not capture diverse perspectives of family caregivers’ unmet needs since 10/13 interviewees were female, however, this represents the average dementia family caregiver profile [1, 5].

## Conclusions

Our findings indicate that, even in the late-stage of dementia, family caregivers continue to experience intertwined information, psychosocial, and self-care needs that are often insufficiently addressed. Family caregivers complained of limited psychological support, poor and fragmented clinical information on disease trajectory, behavioral and psychological symptoms of dementia, and rare discussion about end-of-life care options and the caregiver’s role as a substitute decision-maker. Awareness of community-based supportive services or associations was almost absent. These needs identify potentially modifiable targets for practice through interventions to strengthen dementia understanding and services knowledge, alongside enhanced psychosocial or practical support in daily care.

Mapping community resources can represent the starting point to network for a joint, time-sensitive, and effective answer to dementia family caregivers’ needs. Involving healthcare professionals in such analysis can contribute to better need-based guidance. This mapping can also be useful to inform public health planning and resource allocation.

## Supplementary Information

Supplementary Material 1.

## Data Availability

The datasets used and/or analysed during the current study are available from the corresponding author on reasonable request.

## Abbreviations

NH: Nursing home
PwAD: People with advanced dementia
y: years
