# Supplementary material for: Understanding the family caregivers’ needs in late-stage dementia: a qualitative perspective to inform policy action
Source: BMC Geriatr. 2026 Mar 10;26:534. doi: 10.1186/s12877-026-07201-7 (PMC13085392; doi:10.1186/s12877-026-07201-7)
Supplement: Supplementary file 1 — Supplementary Material 1. [file 12877_2026_7201_MOESM1_ESM.docx]

**Appendix 1.** Report on accordance with the COnsolidated criteria for REporting Qualitative studies (COREQ) guidelines

| **No item** | **Description** |
| --- | --- |
|  |  |
| ***Domain 1: research team and reflexivity*** |  |
| **Personal characteristics** |  |
| 1. Interviewer/ facilitator | G.N. and D.Q. conducted the interviews. |
|  |  |
| 2. Credentials | D.Q. was a master student in nursing sciences; G.N. was a bachelor student in nursing sciences; JMO had a master degree in nursing sciences; S.C. had a PhD and experience in community care; S.G. had PhD in nursing sciences and public health, post-graduate specialization in Bioethics and volunteering experience in nursing home; P.D.G. had MScN and experience in dementia care and end-of-life care; V.D. had MScN and experience in qualitative methodology. |
|  |  |
| 3. Occupation | D.Q. worked as nurse in an Italian hospital; JMO was a research fellow at the University of Torino, Italy; S.G. was assistant professor at the University of Torino, Italy; P.D.G. and V.D. were professors in Nursing Science at the University of Torino, Italy. |
|  |  |
| 4. Gender | G.N, S.C., S.G, and P.D.G. are female; D.Q, J.M.O and V.D. are male. |
| 5. Experience and training | The main investigator S.G. was a nurse trained in dementia care and with experience in research from different areas including quantitative and qualitative research. |
|  |  |
| **Relationship with participants** |  |
| 6. Relationship established | There was no relationship between the interviewers and the participants. No participants were recruited from the nursing homes where S.G volunteered to avoid ethical problems and reporting bias. |
|  |  |
| 7. Participant knowledge of the interviewer | The participants got information that G.N. and D.Q. were master students in nursing from the University of Torino and that the aim of the research was to explore family caregivers’ self-perceived needs in late-stage dementia, primarily in the nursing home context and during the transition into nursing home. When the participants asked, G.N and D.Q told more about their background. |
|  |  |
| 8. Interviewer characteristics | The main interest in the topic was grounded in the well-known priorities of the global action plan on the public health response to dementia 2017-2025 of the World Health Organization. |
|  |  |
| ***Domain 2: study design*** |  |
| **Theoretical framework** |  |
| 9. Methodological orientation | A qualitative descriptive study with inductive content analysis was performed. |
|  |  |
| **Participant selection** |  |
| 10. Sampling | Family caregivers were identified through a variety of strategies: a) nursing home managers; b) the local palliative care service; and c) two local peer support groups for relatives of people with advanced dementia. |
|  |  |
| 11. Method of approach | Overall, the reference person of the local palliative care service, two local peer support groups of dementia caregivers, and 203 nursing home managers were mailed the study protocol and then approached by telephone.  Family caregivers with any degree of kinship with the people with advanced dementia were deemed eligible to participate if: a) they were actively engaged in their relative’s care, at home or in nursing home, based on referrers’ judgement; b) their relative had a Functional Assessment Staging Tool (FAST) score ≥7A (i.e., speech limited to approximately a half-dozen intelligible words or fewer over an average day), or the life expectancy was <6 months, or died from dementia three to twenty months prior to recruitment; and c) were available to participate and provided written informed consent to audiotape their interview. Using these criteria, the nursing home managers, the reference person of the local palliative care service, and the peer support groups identified the family caregivers. Their names with related information for identification were given to the research team who verified that the family caregivers met the inclusion criteria before scheduling the interview. |
| 12. Sample size | In total, 13 family caregivers (11 in nursing home and 2 at home) participated in the study. No family caregivers withdrew from the study. |
|  |  |
| 13. Non-participation | Only 6 nursing home managers (3%) adhered to the study. Four of them found no family caregivers available to participate. No family caregivers were identified by the peer support groups. |
| **Setting** |  |
| 14. Setting of data collection | The data were collected in the North-west of Italy. Interviews took place in the modality (i.e., in-person, telephone) and setting (i.e., interviewee’s home, nursing home) preferred by the family caregiver. |
|  |  |
| 15. Presence of non-participants | No one else beyond the participants and the researcher was present at the interview. |
|  |  |
| 16. Description of sample | The sample is described in the “Methods” section. |
|  |  |
| **Data collection** |  |
| 17. Interview guide | The interview guide was pilot-tested with two family caregivers (data not included in the final dataset) with only minor changes to the final format. |
| 18. Repeat interviews | No repeated interviews were carried out. |
|  |  |
| 19. Audio/visual recordings | All interviews were digitally audio-recorded and stored on a password-protected computer according to the regulations of the Regional ethics committee. |
|  |  |
| 20. Field notes | G.N. and D.Q. made field notes during and after the interviews. The field notes were shared and commented within the research team shortly after each interview to pick up the main features. |
|  |  |
| 21. Duration | Mean duration of interviews was 38 minutes (range 21-80). |
|  |  |
| 22. Data saturation | Thirteen family caregivers were recruited and saturation was reached. |
|  |  |
| 23. Transcripts returned | Participants could review their interview transcript for accuracy. No participants requested copies of transcripts. |
|  |  |
| ***Domain 3: analysis and findings*** |  |
| **Data analysis** |  |
| 24. Number of data coders | G.N, D.Q, and S.G. participated in coding of the data. |
|  |  |
| 25. Description of the coding tree | Table 1 shows an overview of all coding trees. |
|  |  |
| 26. Derivation of themes | Themes were derived from the data. Themes were discussed and agreed on by all the authors. |
|  |  |
| 27. Software | Analysis and coding of the transcripts were aided by the software ATLAS.ti 8. |
|  |  |
| 28. Participant checking | Participants were provided the option of reviewing their interview transcript for accuracy (as explained above). |
|  |  |
| **Reporting** |  |
| 29. Quotations presented | Themes are illustrated by participant quotations that are identified by a code indicating the interviewee’s relationship to the person with advanced dementia, age, and care setting to ensure confidentiality. |
|  |  |
| 30. Data and findings consistent | The presented data are consistent with findings. |
|  |  |
| 31. Clarity of major themes | The major themes are presented in the results and illustrated in Table 1. |
|  |  |
| 32. Clarity of minor themes | The minor themes are presented in the results and illustrated in Table 1. |

**Appendix 2. Interview guide for family caregivers of people with advanced dementia still alive**

**1. Thinking back over the last three months:**

Have there been any changes in your loved one’s health?

Could you tell me about an episode?

Were you prepared for what happened? Why? What were you not expecting?

**2. Thinking about the next weeks or months:**

What do you think might happen to your loved one?

Do you feel prepared to face what might happen in the near future? Yes/No, why?

Do you feel prepared to make decisions about your loved one’s care? Yes/No, why?

**3. There are associations and organizations that offer support to family members of people with dementia.**

Do you know about them?
If yes, which ones do you know? Do you know what services they offer? Do you know how to access them?

**4. Please feel free to add any aspects that you consider important and that were not addressed during the interview.**

**Appendix 3. Interview guide for bereaved family caregivers**

**1. Thinking back to the changes in your loved one’s health during the last month of life**:

Could you tell me about an episode?

Were you prepared for what happened? Yes/No, why? What were you expecting?

**2. Thinking back to your loved one’s health and care in the last month of life:**

What were your main doubts and concerns? Why?

**3. There are associations and organizations that offer support to family members of people with dementia:**

Did you use their services? Yes/No, why?

**4. Please feel free to add any aspects that you consider important and that I have not addressed during the interview.**
